# A Case of Effective Long‐Term Tobramycin Inhalation in a Patient With Bronchiectasis and Refractory *Pseudomonas aeruginosa* Infection

**DOI:** 10.1002/rcr2.70558

**Published:** 2026-03-16

**Authors:** Yasuaki Kotetsu, Nobuyuki Kawaguchi, Maako Nakajima, Ayaka Shiota, Yasuyoshi Washio, Akiko Ishimatsu, Junji Otsuka, Kazuhito Taguchi, Atsushi Moriwaki, Makoto Yoshida

**Affiliations:** ^1^ Department of Respiratory Medicine National Hospital Organization Fukuoka National Hospital Fukuoka Japan

**Keywords:** bronchiectasis, *Pseudomonas aeruginosa*, tobramycin inhalation

## Abstract

Bronchiectasis predisposes patients to refractory airway infections and significantly reduces their quality of life. Here, we report a case of a 78‐year‐old woman with refractory *Pseudomonas aeruginosa* infection complicating bronchiectasis. After *P. aeruginosa* acquired resistance to quinolones, outpatient treatment became difficult, and the patient experienced repeated exacerbations and hospitalisations. Ceftazidime was effective; however, the patient could not be discharged because symptoms worsened immediately after its discontinuation. Initiating nebulised tobramycin for home use markedly improved symptoms and prevented subsequent exacerbation. Furthermore, *P. aeruginosa* isolated from sputum 1 year after initiating nebulised tobramycin regained susceptibility to quinolones. This case suggests that nebulised tobramycin is a viable and effective option for long‐term management of chronic *P. aeruginosa* infection complicating bronchiectasis.

## Graphical abstract

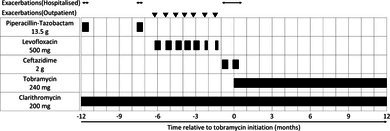

## 1 Introduction

Bronchiectasis provides a milieu for refractory bacterial infections, particularly those caused by *Pseudomonas aeruginosa* [1]. While long‐term macrolide treatment is recommended for patients at high risk of exacerbations, it is not always effective, and many patients continue to experience recurrent episodes. We report a case of bronchiectasis with persistent *P. aeruginosa* infection successfully managed with inhaled tobramycin after long‐term macrolide therapy failed to prevent recurrent exacerbations.

## 2 Case Report

A 78‐year‐old woman with bronchiectasis and a productive cough received long‐term macrolide treatment with clarithromycin (200 mg/day) for 14 years. Although nontuberculous mycobacterial (NTM) pulmonary disease was initially suspected, repeated sputum cultures and bronchoscopy failed to detect NTM; instead, *P. aeruginosa* was consistently identified.

She experienced recurrent exacerbations, each requiring supplemental fluoroquinolone therapy. However, after *P. aeruginosa* acquired resistance to quinolones, outpatient management became increasingly challenging, necessitating multiple hospitalisations for intravenous piperacillin‐tazobactam or ceftazidime therapy.

**FIGURE 1:**
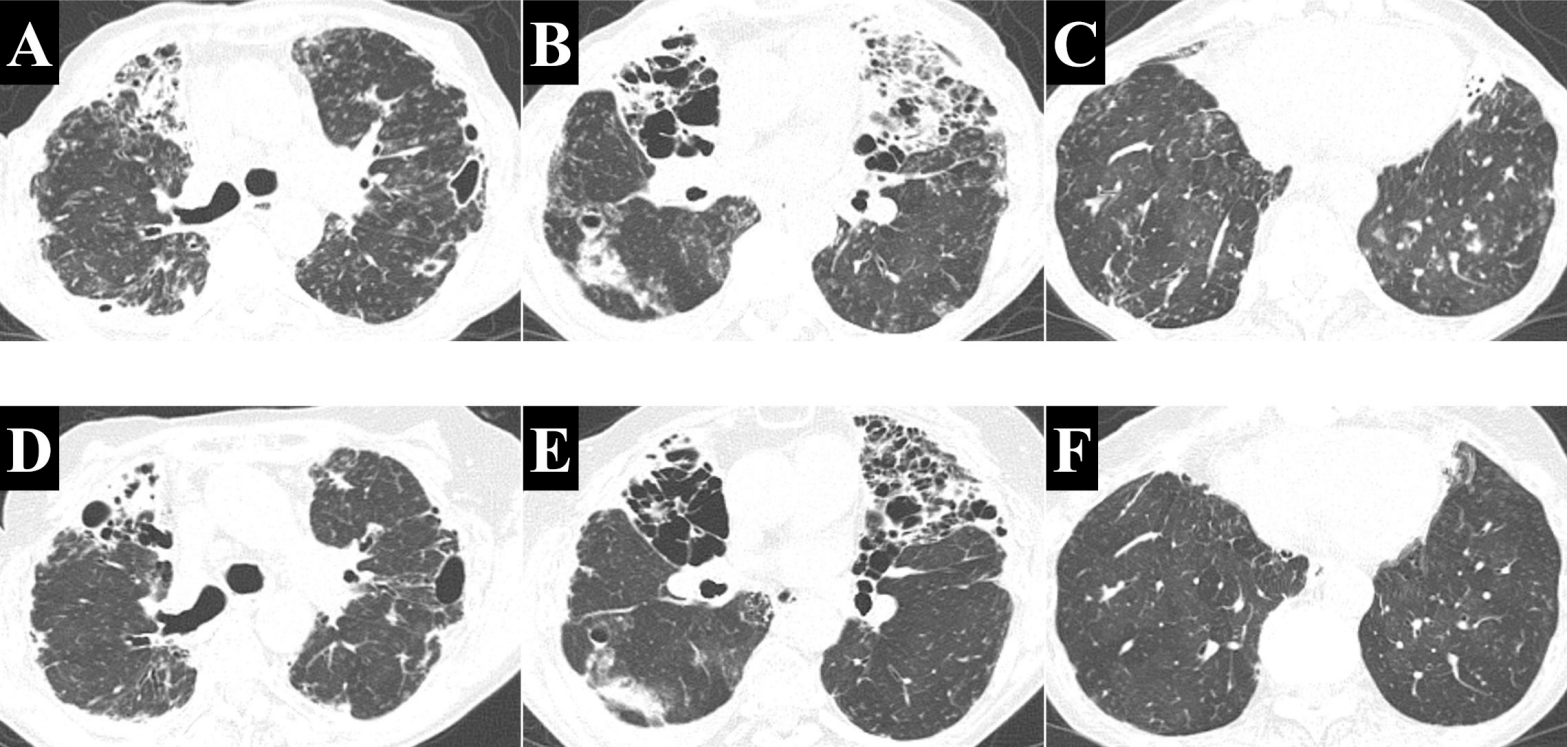
Chest computed tomography (CT) findings in a patient with bronchiectasis and refractory *Pseudomonas aeruginosa* infection. (A–C) CT images at the time of exacerbation. (D–F) CT images 1 year after starting inhaled tobramycin. Centrilobular granular opacities and ground‐glass opacities (GGO) have resolved, and consolidation in the middle lobe and lingula has partially improved.

One week after her last outpatient visit, she presented to the emergency department with dyspnoea and reduced consciousness. Chest computed tomography (CT) revealed new ground‐glass opacities (GGO) and consolidation in both lungs, consistent with an infective exacerbation associated with *P. aeruginosa* (Figure 1A–C). Given a PvCO_2_ of 95.3 Torr, CO_2_ narcosis was identified as the cause of her impaired consciousness.

As she refused intubation, respiratory support was provided using non‐invasive positive pressure ventilation. Concurrently, intravenous ceftazidime (2 g) was initiated with pulmonary rehabilitation for airway clearance. Although the initial response was favourable, symptoms recurred immediately after discontinuation, necessitating repeated courses.

As intravenous therapy could not be discontinued, she could not be discharged. Consequently, nebulised tobramycin was introduced as a feasible at‐home treatment option. As this represents off‐label use, the dosage was set at 120 mg twice daily, the maximum amount permitted under Japanese regulations. Following initiation of tobramycin inhalation, airway clearance efficiency, inflammatory markers, and chest radiographic findings improved rapidly.

**FIGURE 2:**
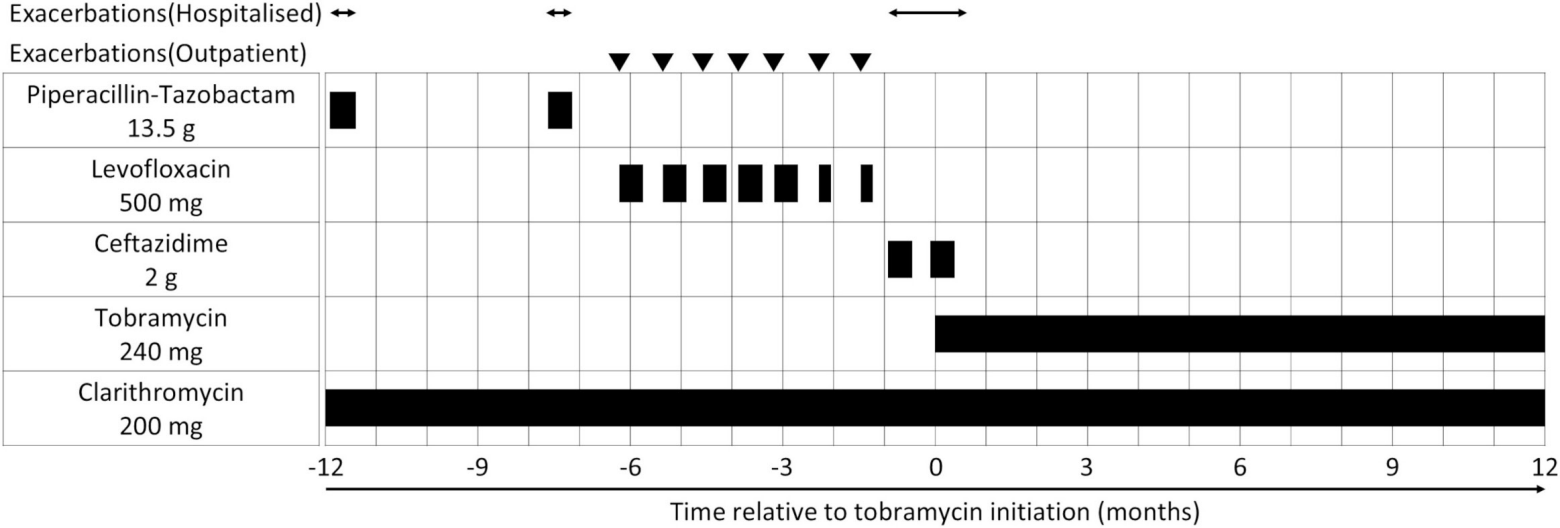
In the year before starting inhaled tobramycin, the patient experienced 10 exacerbations (3 hospitalisations and 7 outpatient visits), with a total of 72 hospital days. The total number of antibiotic days, excluding clarithromycin and tobramycin, was 137 days. In contrast, no exacerbations occurred during the 1‐year period after initiating inhaled tobramycin.

**FIGURE 3:**
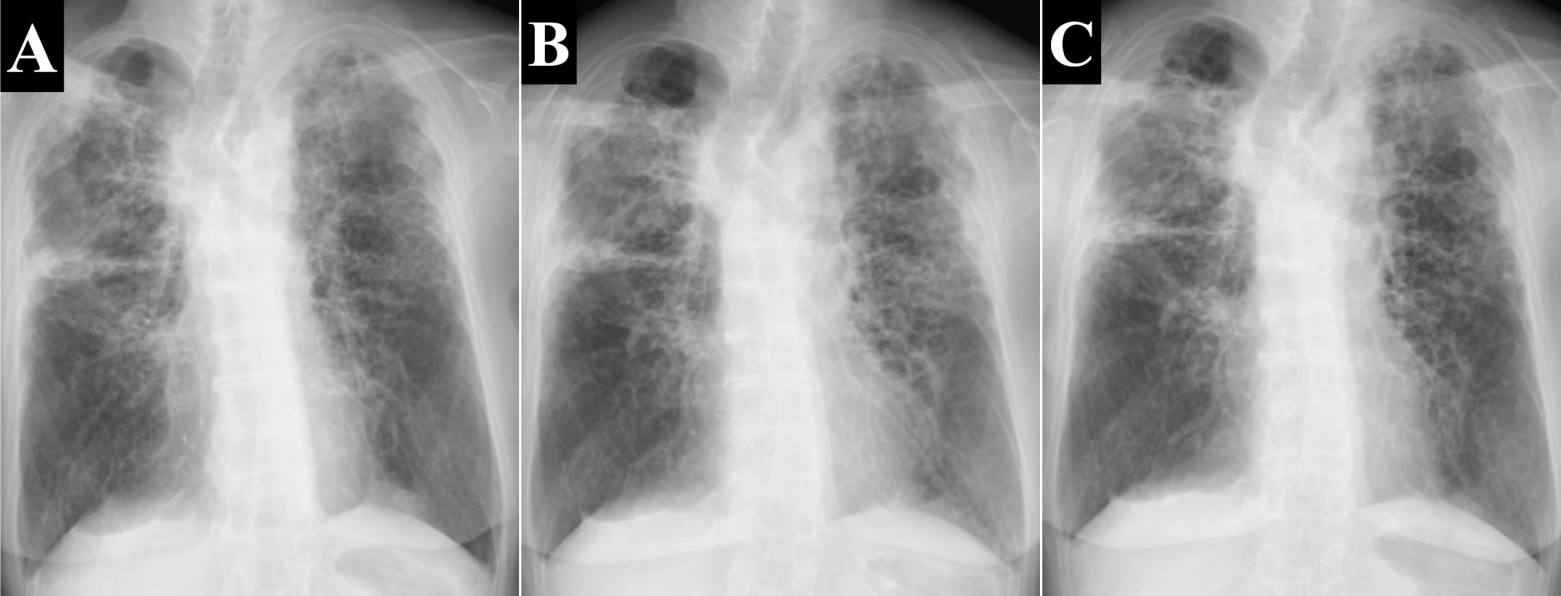
Changes in chest radiographic findings. (A) One year after starting inhaled tobramycin treatment. (B) Three months after discontinuation of inhaled tobramycin, consolidation in the left middle lung field has worsened. (C) One month after resuming inhaled tobramycin, the findings observed during the exacerbation have improved.

After discharge, tobramycin inhalation was continued as a maintenance therapy. To ensure safety, she underwent monthly serum creatinine testing for renal function and routine screening for ototoxicity, including hearing loss, tinnitus, and vertigo. No adverse events occurred during the treatment course. A chest CT scan performed 1 year after initiation of inhaled tobramycin showed resolution of centrilobular granular opacities and GGO, with a partial reduction in consolidation (Figure 1D–F). During tobramycin inhalation, she had no exacerbations and required no additional antimicrobial therapy (Figure 2). After temporary discontinuation of tobramycin, she reported difficulty expectorating sputum within 3 months, with worsening imaging findings (Figure 3A,B). Upon restarting tobramycin inhalation, these findings improved promptly (Figure 3C). Notably, a sputum culture obtained during the exacerbation revealed that *P. aeruginosa* had regained susceptibility to quinolones (Table 1).

**TABLE 1:** Microbiological characteristics of *Pseudomonas aeruginosa* isolates.

Timing of sputum collection	> 1 year before tobramycin initiation	Within 1 year before tobramycin initiation	1 year after tobramycin initiation
Phenotype	Mucoid	Mucoid	Rough
AST method	Disk diffusion	Disk diffusion	VITEK 2 Compact
Levofloxacin	S (MIC N/A)	R (MIC N/A)	S (MIC 0.5)

*Note:* For the mucoid phenotype, the disk diffusion method was used because the automated VITEK 2 Compact system (bioMérieux, Marcy l'Étoile, France) is not validated for slow‐growing bacteria such as mucoid *Pseudomonas aeruginosa* . Consequently, MIC values were not determined (N/A) for these samples.

Abbreviations: AST, antimicrobial susceptibility testing; MIC, minimum inhibitory concentration (μg/mL); N/A, not applicable; R, resistant; S, susceptible.

## 3 Discussion

The treatment of *P. aeruginosa* infection associated with bronchiectasis is difficult owing to limited therapeutic options and poor disease control. In contrast, for *P. aeruginosa* infection associated with cystic fibrosis, inhaled tobramycin is an established therapy shown to improve lung function, reduce *P. aeruginosa* density in sputum, and decrease hospitalisation risk [2]. In this case, we successfully managed *P. aeruginosa* infection complicating bronchiectasis over the long term using low‐dose tobramycin inhalation without adverse effects.

A double‐blind randomised placebo‐controlled trial evaluated inhaled tobramycin efficacy in bronchiectasis with *P. aeruginosa* infection [3]. Compared with the placebo group, the tobramycin inhalation group demonstrated a significant reduction in *P. aeruginosa* density, improvement in the Quality‐of‐Life Bronchiectasis Respiratory Symptoms score, and increased microbiological eradication. Both groups exhibited similar safety profiles, with no significant differences in adverse or serious adverse events.

The 2025 European Respiratory Society clinical practice guidelines for adult bronchiectasis strongly recommend long‐term inhaled antibiotics for patients with chronic *P. aeruginosa* infection at high risk of exacerbations despite standard care [1]. However, specific agents and optimal dosages have not been established, and the ideal regimen remains controversial. While many clinicians prescribe inhaled tobramycin at 300 mg twice daily—extrapolating from cystic fibrosis protocols—our patient received 120 mg twice daily in accordance with Japanese regulations. Despite this lower dose, a significant clinical effect was achieved. This suggests that inhaled tobramycin doses could be optimised based on individual patient factors such as body habitus and severity of structural lung abnormalities.

Meta‐analyses of inhaled antibiotic therapy for bronchiectasis complicated by *P. aeruginosa* infection have demonstrated that long‐term treatment is associated with an increased risk of resistance to the administered agents [4]. Inhaled tobramycin has been associated with an increase in minimum inhibitory concentration from baseline [3]. In our case, sputum *P. aeruginosa* isolates showed no tobramycin resistance after 1 year, whereas quinolone susceptibility was concurrently restored. Generally, antibiotic‐resistant strains incur higher fitness costs than susceptible strains. Consequently, discontinuing a specific antimicrobial agent may allow susceptible strains to outcompete resistant strains through natural selection. However, in quinolone‐resistant *P. aeruginosa* , simple withdrawal may not restore susceptibility because bacteria can recover their growth rates by acquiring compensatory mutations [5]. Our findings suggest that using a different antibiotic class, such as aminoglycosides, may facilitate restoration of quinolone susceptibility. Given the difficulty of eradicating *P. aeruginosa* in patients with bronchiectasis, quinolone susceptibility was considered restored within the same strain rather than reflecting strain replacement before and after tobramycin inhalation. However, this hypothesis requires confirmation by genomic typing, which is a limitation of this study.

In conclusion, this case suggests that inhaled tobramycin is not only an effective long‐term management strategy for bronchiectasis with *P. aeruginosa* infection but may also facilitate restoration of quinolone susceptibility in resistant strains.

## Author Contributions

Y.K. wrote the original draft. M.Y. wrote the review and editing. N.K., M.N., A.S., Y.W., A.I., J.O., K.T. and A.M. contributed to the management of the patient. All authors have approved the final manuscript.

## Ethics Statement

The use of inhaled tobramycin for non‐cystic fibrosis bronchiectasis in this case was conducted in compliance with Japanese regulations. Written informed consent for this off‐label treatment was obtained from the patient prior to its administration.

## Consent

The authors declare that written informed consent was obtained for the publication of this manuscript and accompanying images using the consent form provided by the Journal.

## Conflicts of Interest

The authors declare no conflicts of interest.

## Data Availability

The data that support the findings of this study are available from the corresponding author upon reasonable request.

## References

[1] J. D. Chalmers , C. S. Haworth , P. Flume , et al., “European Respiratory Society Clinical Practice Guideline for the Management of Adult Bronchiectasis,” European Respiratory Journal 66 (2025): 2501126. 41016738 10.1183/13993003.01126-2025

[2] B. W. Ramsey , M. S. Pepe , J. M. Quan , et al., “Intermittent Administration of Inhaled Tobramycin in Patients With Cystic Fibrosis,” New England Journal of Medicine 340 (1999): 23–30. 9878641 10.1056/NEJM199901073400104

[3] W. J. Guan , J. F. Xu , H. Luo , et al., “A Double‐Blind Randomized Placebo‐Controlled Phase 3 Trial of Tobramycin Inhalation Solution in Adults With Bronchiectasis With *Pseudomonas aeruginosa* Infection,” Chest 163 (2023): 64–76. 35863486 10.1016/j.chest.2022.07.007

[4] R. Cordeiro , H. Choi , C. S. Haworth , and J. D. Chalmers , “The Efficacy and Safety of Inhaled Antibiotics for the Treatment of Bronchiectasis in Adults: Updated Systematic Review and Meta‐Analysis,” Chest 166 (2024): 61–80. 38309462 10.1016/j.chest.2024.01.045 PMC11251083

[5] E. Kugelberg , S. Löfmark , B. Wretlind , and D. I. Andersson , “Reduction of the Fitness Burden of Quinolone Resistance in *Pseudomonas aeruginosa* ,” Journal of Antimicrobial Chemotherapy 55 (2005): 22–30. 15574475 10.1093/jac/dkh505

